# Supplementary material for: Unique Habitat of Karst Tiankengs Changes the Taxonomy and Potential Metabolism of Soil Microbial Communities
Source: Microbiol Spectr. 2023 Jan 17;11(1):e02316-22. doi: 10.1128/spectrum.02316-22 (PMC9927240; doi:10.1128/spectrum.02316-22)
Supplement: Supplemental file 1 — Supplemental material. Download spectrum.02316-22-s0001.pdf, PDF file, 1.1 MB [file spectrum.02316-22-s0001.pdf]

Supplementary Information for

The unique habitat of karst tiankengs changes the taxonomy and potential metabolism of soil microbial communities

Cong Jiang[a], Hui Zeng[a*],

[a] School of Urban Planning and Design, Peking University Shenzhen Graduate School, Peking University, Shenzhen, China

*** Correspondence:**
Corresponding Author: Hui Zeng
zengh@pkusz.edu.cn

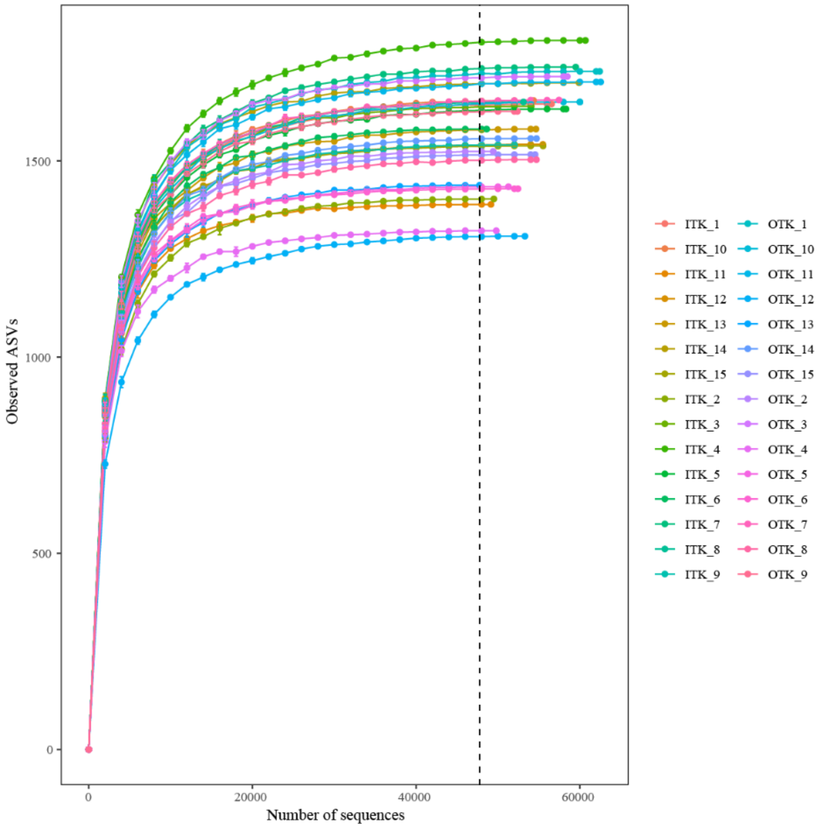

Fig. S1 Rarefaction curves of microbial communities inside and outside karst tiankeng.

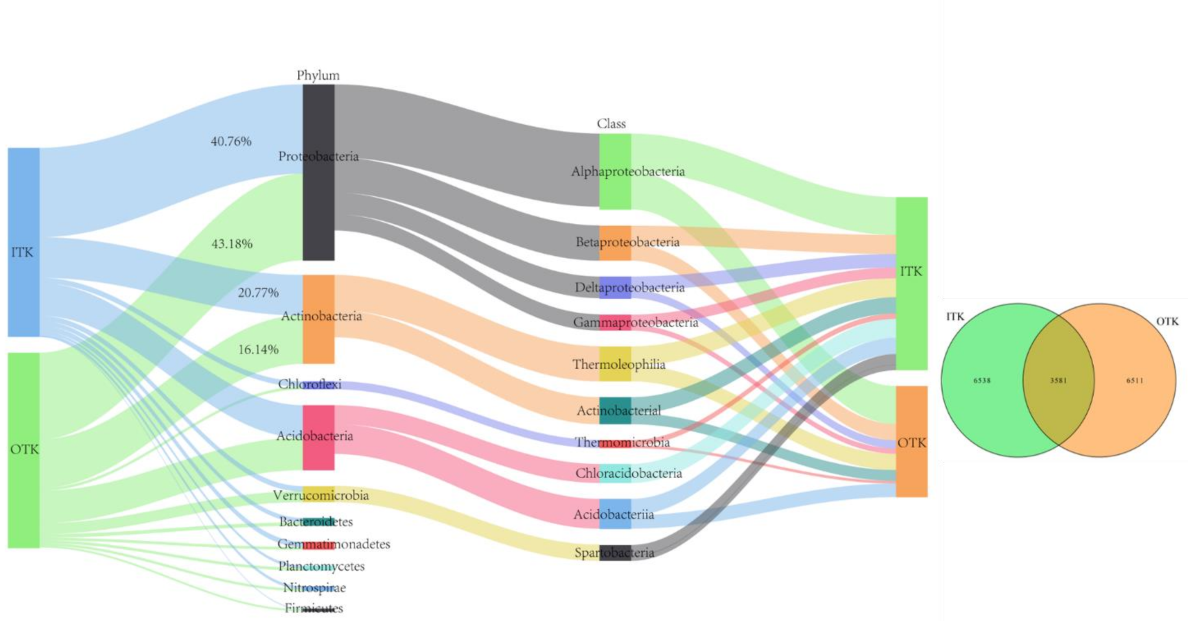

Fig. S2 The composition of microbial communities inside and outside karst tiankeng, and represented in Sanchi diagrams.

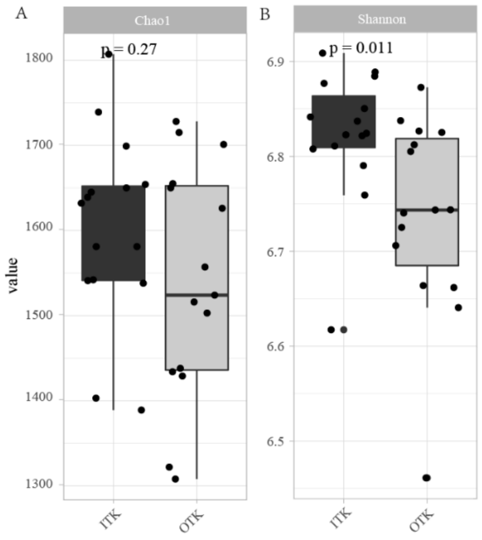

Fig. S3 Alpha-diversity indices of microbial communities inside and outside karst tiankeng **(A)**. Chao1 index **(B)**. Shannon index.

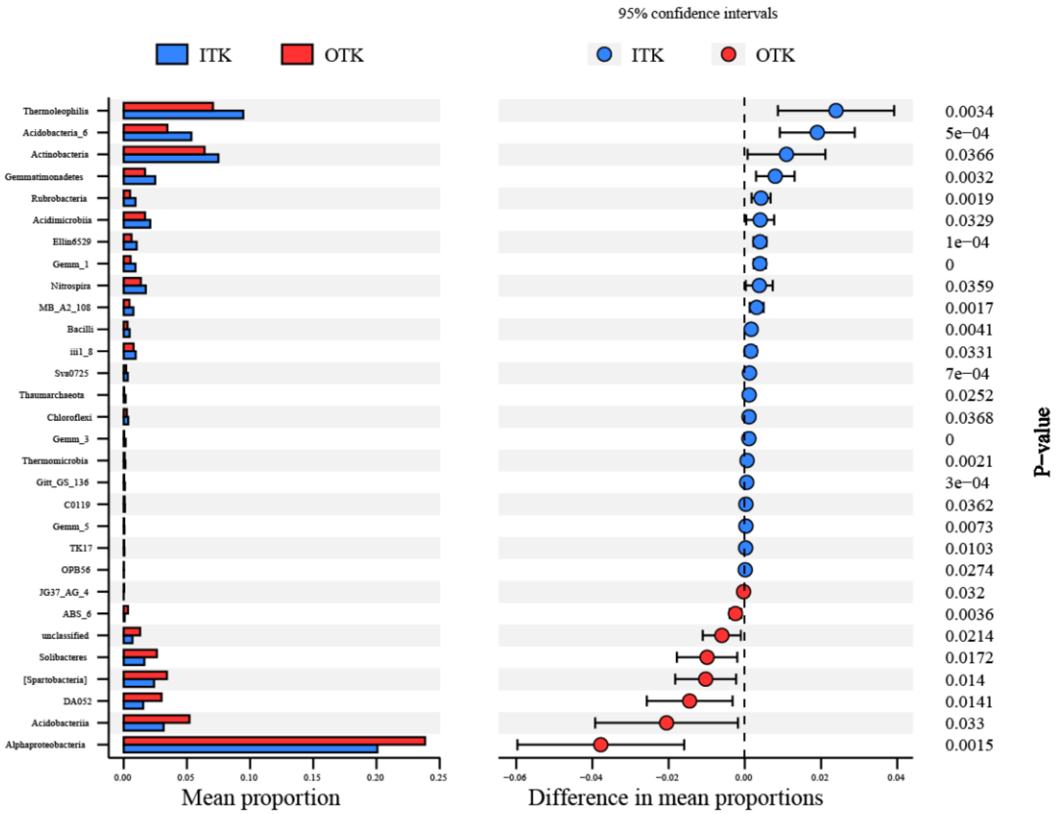

Fig. S4 STAMP analysis of microbial communities at classes level inside and outside karst tiankeng ($P < 0.05$).

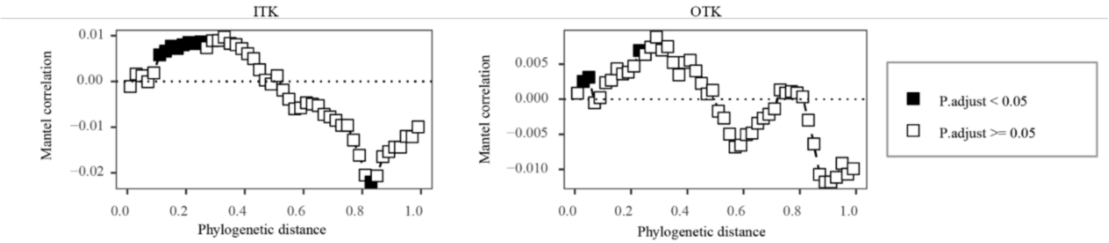

Fig. S5 Mantel correlograms between the phylogenetic distances and pairwise of OTU niche distances inside and outside karst tiankeng soil. Black points represent significant phylogenetic signals ($P < 0.05$).

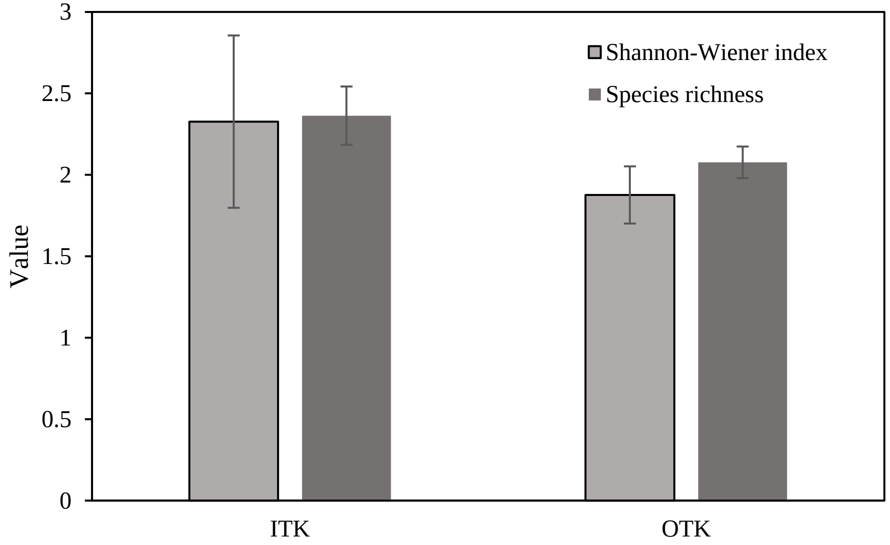

Fig. S6 The diversity and richness of plant communities at inside and outside karst tiankeng.

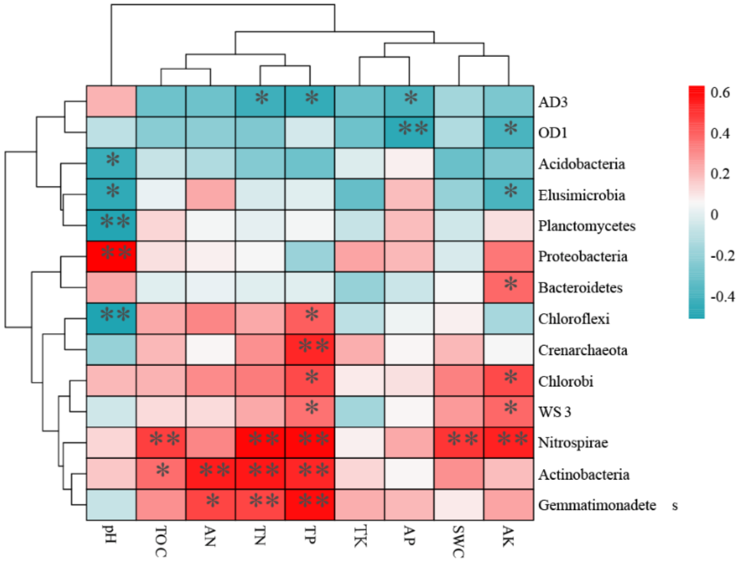

Fig. S7 Spearman correlation between soil physiochemical properties and major bacterial phyla. The color represents the value of the spearman correlation coefficient; red indicates a positive correlation, and blue indicates a negative correlation. * indicates a significant correlation at $P < 0.05$; ** indicates a significant correlation at $P < 0.01$.

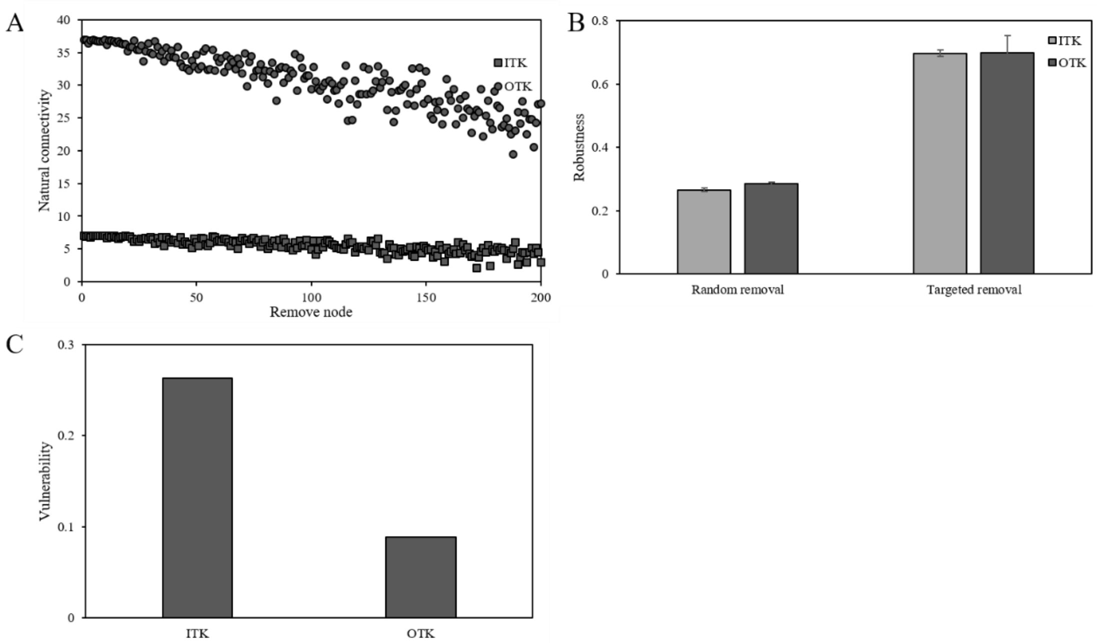

Fig. S8 The natural connectivity **(A)**, robustness **(B)** and vulnerability **(C)** of microbial network inside and outside karst tiankeng.

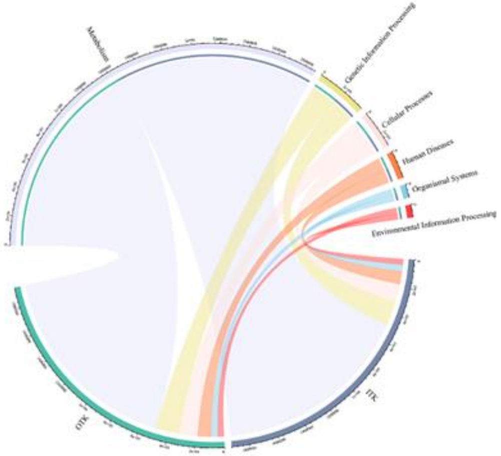

Fig. 9 The potential function of microbial community inside and outside karst tiankeng.

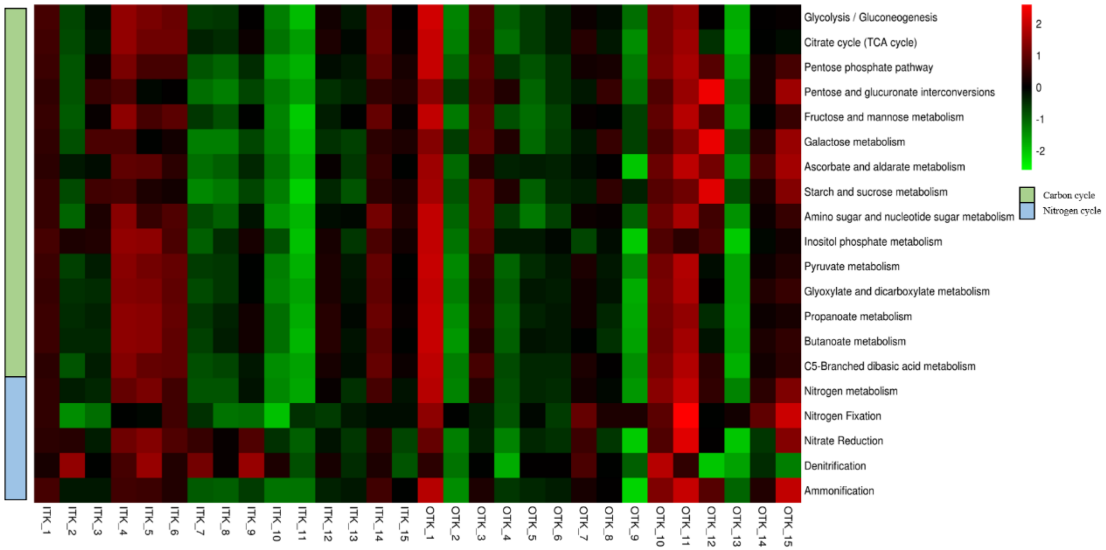

Fig. S10 The abundance of genes associated with C and N cycle inside and outside karst tiankeng.

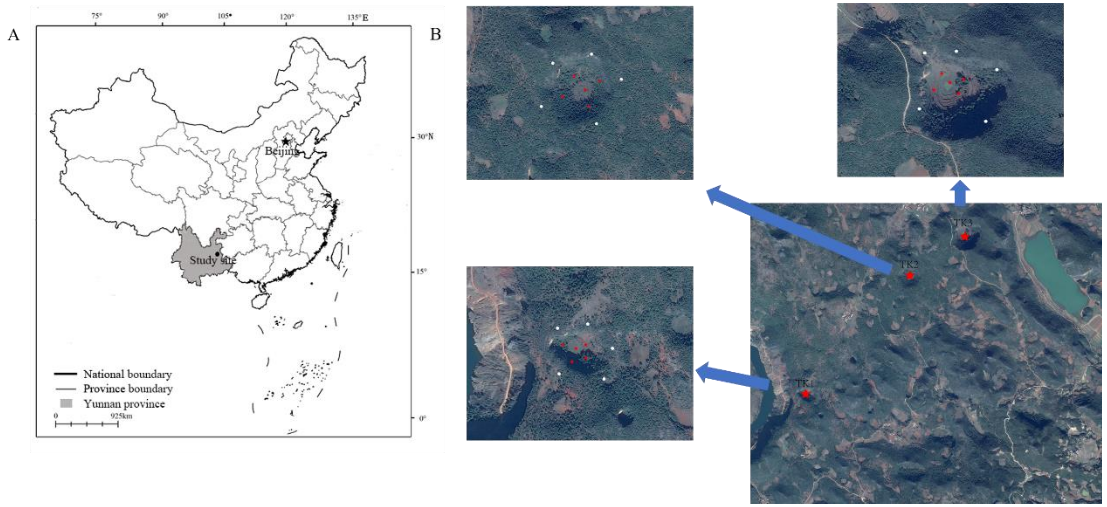

Fig. S21. Location of study site on the map of China **(A)** Sampling sites at Zhanyi tiankeng group (TK1, Bajiaxiantang tiankeng; TK2, Shaojiaxiantang Tiankeng; TK3, Shenxiantang tiankeng) **(B)**. The red pentacle represents the inside tiankeng sites and the white pentacle represents the outside tiankeng sites.

Table S1. PERMANOVA, ANOSIM and MRPP analysis of microbial communities inside (ITK) and outside (OTK) karst tiankeng.

	**PERMANOVA**		**ANOSIM**		**MRPP**	
	r^2	P	r	P	δ	P
ITK vs. OTK	0.12	0.001**	0.35	0.001**	0.53	0.001**

Table S2. Nearest taxon index (NTI) values of microbial communities inside and outside karst tiankeng.

**ITK**		**OTK**	
Site name	NTI	Site name	NTI
ITK_1	15.89	OTK_1	15.82
ITK_2	9.92	OTK_2	14.40
ITK_3	14.60	OTK_3	15.25
ITK_4	14.71	OTK_4	14.47
ITK_5	12.64	OTK_5	14.22
ITK_6	14.80	OTK_6	15.35
ITK_7	14.47	OTK_7	14.89
ITK_8	15.21	OTK_8	14.05
ITK_9	14.86	OTK_9	13.04
ITK_10	14.47	OTK_10	15.17
ITK_11	16.13	OTK_11	14.46
ITK_12	16.31	OTK_12	12.95
ITK_13	15.15	OTK_13	12.24
ITK_14	16.27	OTK_14	14.20
ITK_15	16.05	OTK_15	12.86
Mean	14.76	Mean	14.22

Table S3. Pearson correlations between soil microbial taxonomic diversity (Shannon index) and soil physiochemical properties inside and outside karst tiankeng.

	**Microbial taxonomic profile**	
	r	p
SWC	0.31	0.097
TOC	0.36	0.051
TN	0.45	0.012
TP	0.48	0.007
TK	0.16	0.390
AK	0.40	0.027
AP	0.21	0.258
AN	0.38	0.039
pH	-0.01	0.960

Table S4. Topological indices of keystone taxa in the networks of inside and outside karst tiankeng.

		**Name**	**node.degree**	**node.betw**	**node.stress**	**node.evcent**	**CC**	**No. module**	***Zi***	***Pi***
ITK	Modules hubs	p__Actinobacteria; c__Actinobacteria; o__Actinomycetales; f__Pseudonocardiaceae; g__Pseudonocardia	35	6261.08	54923	0.02	0.03	3	4.74	0.06
		p__Firmicutes; c__Bacilli; o__Bacillales	16	4091.21	36879	0.05	0.03	4	4.19	0.40
		p__Acidobacteria; c__Acidobacteriia; o__Acidobacteriales; f__Koribacteraceae	26	2887.39	36139	0.02	0.01	3	3.45	0.00
		p__Proteobacteria; c__Alphaproteobacteria; o__Rhizobiales; f__Phyllobacteriaceae; g__Mesorhizobium	15	2503.92	17780	0.21	0.37	2	2.71	0.12
		p__Proteobacteria; c__Betaproteobacteria; o__Burkholderiales; f__Comamonadaceae; g__Hydrogenophaga	17	1493.08	11769	0.23	0.32	1	2.70	0.00
		p__Proteobacteria;	18	1693.05	9459	0.20	0.2	1	2.7	0.1

	c__Alphaproteobacteria; o__Sphingomonadales					6		0	0
	p__Acidobacteria; c__Chloracidobacteria; o__RB41	22	1879.06	20165	0.01	0.00	3	2.65	0.09
	p__Actinobacteria; c__Thermoleophilia; o__Solirubrobacterales	9	1974.58	20322	0.01	0.08	0	2.54	0.49
	p__Proteobacteria; c__Alphaproteobacteria; o__Rhizobiales; f__Hyphomicrobiaceae; g__Rhodoplanes	6	653.62	6551	0.00	0.07	0	2.54	0.00
Connectors	p__Bacteroidetes; c__Cytophagia; o__Cytophagales; f__Cytophagaceae	5	1021.41	6719	0.04	0.00	5	-0.26	0.72
	p__Proteobacteria; c__Gammaproteobacteria; o__Xanthomonadales; f__Xanthomonadaceae; g__Lysobacter	6	1247.90	10443	0.03	0.00	5	0.56	0.67
	p__Proteobacteria; c__Alphaproteobacteria; o__Rhodospirillales; f__Rhodospirillaceae; g__Skermanella	12	5685.48	65576	0.08	0.08	1	0.15	0.67
	p__Proteobacteria;	18	3227.20	22343	0.20	0.2	2	1.0	0.6

c__Alphaproteobacteria; o__Rhodospirillales; f__Rhodospirillaceae					2		1	6
p__Proteobacteria; c__Alphaproteobacteria; o__Rhizobiales	5	262.10	1850	0.05	0.00	2	-0.69	0.64
p__Proteobacteria; c__Alphaproteobacteria; o__Rhizobiales; f__Bradyrhizobiaceae; g__Bradyrhizobium	11	3235.37	15349	0.08	0.15	2	0.16	0.63
p__Proteobacteria; c__Alphaproteobacteria; o__Rhizobiales; f__Hyphomicrobiaceae; g__Pedomicrobium	4	775.23	6814	0.02	0.00	5	-0.26	0.63
p__Verrucomicrobia; c__Spartobacteria; o__Chthoniobacterales; f__Chthoniobacteraceae; g__Candidatus_Xiphinematobacter	4	333.18	1612	0.01	0.00	0	-0.33	0.63
p__Gemmatimonadetes; c__Gemm_3	4	138.70	910	0.05	0.33	4	-0.74	0.63
p__Proteobacteria;	4	229.43	3174	0.06	0.3	4	-	0.6

		c__Betaproteobacteria; o__Burkholderiales; f__Oxalobacteraceae; g__Cupriavidus					3		0.74	3
OTK	Modules hubs	p__Verrucomicrobia; c__Spartobacteria; o__Chthoniobacterales; f__Chthoniobacteraceae; g__DA101	40	4660.40	35091	0.28	0.18	2	3.73	0.19
		p__Planctomycetes; c__Planctomycetia; o__Gemmatales; f__Isosphaeraceae	29	4878.18	67889	0.09	0.07	0	3.49	0.19
		p__Acidobacteria; c__DA052; o__Ellin6513	27	4525.79	68454	0.09	0.11	0	3.31	0.14
		p__Proteobacteria; c__Betaproteobacteria; o__Burkholderiales; f__Alcaligenaceae	7	1714.97	20248	0.00	0.00	5	3.08	0.24
		p__Proteobacteria; c__Gammaproteobacteria; o__Pseudomonadales; f__Pseudomonadaceae; g__Pseudomonas; s__veronii	35	1816.42	33091	0.30	0.25	2	3.06	0.20
		p__Acidobacteria; c__Acidobacteriia;	6	1979.74	17677	0.00	0.07	1	3.02	0.00

	o__Acidobacteriales; f__Koribacteraceae; g__Candidatus_Koribacter								
	p__Proteobacteria; c__Gammaproteobacteria; o__Xanthomonadales; f__Xanthomonadaceae; g__Lysobacter; s__yangpyeongensis	7	1315.89	15829	0.00	0.14	4	2.63	0.00
Connectors	p__Gemmatimonadetes; c__Gemmatimonadetes	3	20.82	248	0.02	0.33	3	-1.11	0.67
	p__Actinobacteria; c__Thermoleophilia; o__Gaiellales; f__Gaiellaceae	3	32.97	388	0.01	0.00	3	-1.11	0.67
	p__Proteobacteria; c__Alphaproteobacteria; o__Rhodospirillales; f__Rhodospirillaceae; g__Reyranella; s__massiliensis	5	295.31	1658	0.01	0.10	1	0.00	0.64
	p__Proteobacteria; c__Betaproteobacteria; o__SC_I_84	4	329.61	4268	0.01	0.17	1	-0.76	0.63

CC, Node Clustering Coefficient; Node betw, Node betweeness centrality.

Table S5. The morphological characteristics and Vegetation characteristics of three tiankengs

	**Morphological characteristics**			**Vegetation characteristics**		
	**Depth-to-width ratio**	**Damage degree of tiankeng wall**	**Number of toppling slopes**	**Shannon-Wiener index**	**Species richness**	**Main plant**
Shenxiantang	0.43	42%	3	2.25	2.56	*Myrsine africana* Linn.<br>*Debregeasia orientalis* C. J. Chen<br>*Swida oblonga*
Bajiaxiantang	0.35	39%	2	2.89	2.32	*Quercus variabilis*<br>*Alangium chinense*<br>*Cyclobalanopsis glauca*
Shaojiaxiantang	1.16	78%	4	1.84	2.21	*Swida oblonga*<br>*Cyclobalanopsis glauca*
